# The Impact of COVID-19 on Treatment Practices for Patients With Early Breast Cancer: A Cross-Sectional Study From a Large Cancer Center in Italy

**DOI:** 10.1093/oncolo/oyad255

**Published:** 2023-09-12

**Authors:** Fabio Girardi, Sabrina Marini, Francesca Porra, Sonia Carpentieri, Alberto Marchet, Tania Saibene, Marcello Lo Mele, Tommaso Giarratano, Carlo Alberto Giorgi, Eleonora Mioranza, Cristina Falci, Giovanni Faggioni, Francesca Caumo, Gaia Griguolo, Maria Vittoria Dieci, Valentina Guarneri

**Affiliations:** Division of Medical Oncology 2, Veneto Institute of Oncology IOV-IRCCS, Padua, Italy; Division of Medical Oncology 2, Veneto Institute of Oncology IOV-IRCCS, Padua, Italy; Department of Surgery, Oncology and Gastroenterology, University of Padua, Padua, Italy; Division of Medical Oncology 2, Veneto Institute of Oncology IOV-IRCCS, Padua, Italy; Department of Surgery, Oncology and Gastroenterology, University of Padua, Padua, Italy; Division of Breast Surgery, Veneto Institute of Oncology IOV-IRCCS, Padua, Italy; Division of Breast Surgery, Veneto Institute of Oncology IOV-IRCCS, Padua, Italy; Division of Breast Surgery, Veneto Institute of Oncology IOV-IRCCS, Padua, Italy; Division of Surgical Pathology, Azienda Ospedale-Università Padova, Padua, Italy; Division of Medical Oncology 2, Veneto Institute of Oncology IOV-IRCCS, Padua, Italy; Division of Medical Oncology 2, Veneto Institute of Oncology IOV-IRCCS, Padua, Italy; Division of Medical Oncology 2, Veneto Institute of Oncology IOV-IRCCS, Padua, Italy; Division of Medical Oncology 2, Veneto Institute of Oncology IOV-IRCCS, Padua, Italy; Division of Medical Oncology 2, Veneto Institute of Oncology IOV-IRCCS, Padua, Italy; Division of Breast Imaging, Veneto Institute of Oncology IOV-IRCCS, Padua, Italy; Division of Medical Oncology 2, Veneto Institute of Oncology IOV-IRCCS, Padua, Italy; Department of Surgery, Oncology and Gastroenterology, University of Padua, Padua, Italy; Division of Medical Oncology 2, Veneto Institute of Oncology IOV-IRCCS, Padua, Italy; Department of Surgery, Oncology and Gastroenterology, University of Padua, Padua, Italy; Division of Medical Oncology 2, Veneto Institute of Oncology IOV-IRCCS, Padua, Italy; Department of Surgery, Oncology and Gastroenterology, University of Padua, Padua, Italy

**Keywords:** COVID-19, breast cancer, cancer care, treatment delay

## Abstract

**Introduction:**

The Coronavirus Disease 2019 (COVID-19) has disrupted health services worldwide. The evidence on the impact of the pandemic on cancer care provision, however, is conflicting. We aimed to audit the management of patients diagnosed with early breast cancer (EBC) during the pandemic in a large, tertiary-level cancer center in Italy.

**Methods:**

We conducted a cross-sectional study to track the route to first treatment for patients diagnosed with EBC during 2019, 2020, and 2021. We abstracted data for all consecutive patients referred to the Veneto Institute of Oncology (Padua, Italy). We defined as point of contact (POC) the date of the first consultation with a breast cancer specialist of the breast unit. First treatment was defined as either upfront surgery or neoadjuvant chemotherapy (NACT).

**Results:**

We reviewed medical records for 878 patients for whom an MDT report during 2019-2021 (April through June) was available. Of these, 431 (49%) were eligible. The proportion of screen-detected tumors was larger in 2019 and 2021 than in 2020 (59%). Conversely, the proportion of screen-detected tumors was offset by the proportion of palpable tumors in 2020 (*P* = .004). Distribution of tumor and nodal stage was unchanged over time, but in situ tumors were slightly fewer in 2020 than in 2019 or 2021. The adjusted odds ratio for treatment delay (45 days or more) was 0.87 for 2020 versus 2019 (95% CI, 0.5-1.53) and 0.9 for 2021 versus 2019 (95% CI, 0.52-1.55).

**Conclusions:**

There was no evidence for major changes in the management of patients with EBC during 2019-2021 and no treatment delays were observed. Our findings suggest that more women presented with palpable nodules at diagnosis, but the stage distribution did not change over time. Validation on a larger cohort of patients is warranted to robustly assess the impact of the COVID-19 pandemic on treatment practices for patients with EBC.

## Teaser

This article evaluates the impact on the distribution of disease parameters and time to care of patients with breast cancer during the SARS-CoV2 pandemic, using data from an audit of the management of patients diagnosed with early breast cancer during the pandemic in a large cancer center in Italy.

**Implications for Practice**

In this study, granular patient- and treatment-related data were used to track the route to the first curative treatment for patients diagnosed with early breast cancer (EBC) during COVID-19. All eligible patients for the relevant catchment area were ascertained. The authors could not find major changes in treatment practices or in the stage distribution for patients with EBC during the pandemic. The study was conducted in a tertiary-level cancer center with dedicated cancer services, but it cannot be ruled out that delays may have occurred in primary and secondary care settings. Validation of these findings in larger, population-based studies is warranted. This study may serve as a platform to audit quality of cancer care during the pandemic at regional and national level and to inform future preparedness plans.

## Introduction

The coronavirus disease 2019 (COVID-19) pandemic resulted in a severe disruption of healthcare services. To control the spread of SARS-CoV2, governments around the world enforced stay-at-home measures. In Italy, a lockdown was in place from 9 March to 4 May, 2020.

International agencies such as the Centers for Disease Control and Prevention in the US and the European Centre for Disease Prevention and Control issued recommendations for the reorganization of healthcare delivery, including postponement of all elective procedures and suspension of breast cancer screening programs.^1-4^ In the Veneto region, in North-Eastern Italy, where this study was conducted, breast cancer screening was suspended in March and April 2020.^5,6^

In this context, international associations such as the European Society of Medical Oncology (ESMO) and the American Society of Clinical Oncology (ASCO), provided guidance for mitigating the impact of COVID-19 on the management of breast cancer patients, by prioritizing selected treatments and procedures both in the adjuvant and metastatic setting.^3,7-9^

The acute disruption of healthcare led to growing concern that delays in diagnosis and care would shift the stage distribution of breast cancer patients toward more advanced stages or impair cancer survival. However, the available evidence on the impact of the pandemic on cancer care provision is conflicting. Some reports found that management for patients diagnosed with early breast cancer (EBC) during the pandemic did not differ from pre-pandemic practices; others suggested that delays in breast cancer surgery may have occurred.^10,11^

The Veneto Institute of Oncology is a tertiary-level comprehensive cancer center covering a population of nearly 5 million people. The breast unit, among the busiest in the country, provides for a catchment area of one million people and receives referrals from primary and secondary-level hospitals in the region and beyond. We evaluated the care pathway in patients referred to the breast unit during the quarters of April-June 2019, 2020, and 2021.

The aim of the study is to evaluate the impact on the distribution of disease parameters and time to care for patients with breast cancer during the COVID-19 pandemic. We aimed to audit the management of patients diagnosed with EBC during the pandemic in a large cancer center in Italy.

## Patients and Methods

We conducted a cross-sectional study to track the route to first treatment for patients diagnosed with EBC during 2019, 2020, and 2021. We considered all consecutive patients treated at the Veneto Institute of Oncology (Padua, Italy).

We chose the 3-month period from April to June because in 2020 it was when the COVID-19 pandemic first acutely hit, leading to contingency plans that included prioritizing acute care, repurposing of services offering deferrable treatments and closure of cancer screening programs. We analyzed the same period for 2019 and 2021 to minimize confounding.

We considered patients with a first point of contact (POC) in the 6 months preceding the multidisciplinary (MDT) meeting and initiating a treatment within 6 months from the POC. We defined as point of contact (POC) the date of the first consultation with a breast cancer specialist of the breast unit. First treatment was defined as either upfront surgery or neoadjuvant chemotherapy (NACT). The time to first treatment was defined as the interval between the first POC and the first treatment. We used the median time to first treatment in 2019 to define the threshold for treatment delay. We abstracted routinely collected data from electronic medical records.

We defined the following variables: age, type of presentation, type of POC, tumor and nodal stage, tumor type, and treatment type. Differences between categorical variables were tested for significance using the chi-squared statistics. The risk of treatment delay was assessed using a multivariable logistic regression model.

The requirement for an ethics approval was waived as all data had been routinely collected as part of the electronic medical records. Patients formally consented to personal data collection and handling, in compliance with data protection regulations.

## Results

We reviewed electronic medical records for 878 patients for whom an MDT report during 2019-2021 (April through June) was available. Of these, 431 (49%) were eligible: 144 in 2019, 127 in 2020 and 150 in 2021. Median age at first POC was 61 years in 2010, 60 in 2020, and 63 in 2021.

**Figure 1.**
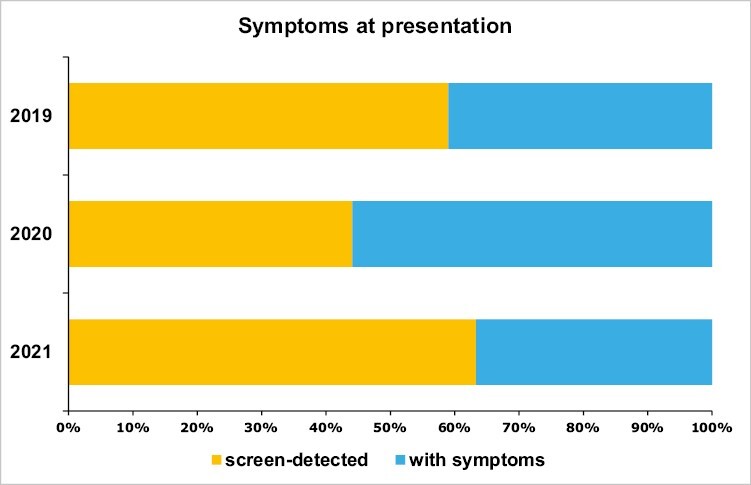
Distribution by symptoms at presentation.

The proportion of patients presenting with tumors detected at screening were 59% in 2019, 44% in 2020, and 63% in 2021. The proportion of patients with screen-detected tumors was offset by the proportion of palpable tumors only in 2020. These differences were statistically significant (chi-square test 11.12, *P* = .004; Table 1; Fig. 1).

**Table 1.** Characteristics of the patient population.

	Apr-Jun 2019	Apr-Jun 2020	Apr-Jun 2021	
Number of patients	144	127	150	
Age (median)	60	63	63	
Presentation				*P* = .004
With symptoms	85 (59)^*^	56 (44)	95 (63)	
Without symptoms	59 (41)	71 (56)	55 (37)	
POC^†^ type				*P* = .15
Breast radiologist	51 (35)	29 (23)	55 (37)	
Breast surgeon	88 (61)	92 (72)	87 (58)	
Medical oncologist	2 (1)	4 (3)	6 (4)	
Other	3 (3)	2 (2)	2 (1)	
Tumour stage				*P* = .88
T0	10 (7)	7 (6)	14 (9)	
T1	75 (52)	73 (57)	81 (54)	
T2	46 (32)	37 (29)	41 (27)	
T3-T4	13 (9)	9 (7)	13 (9)	
Unknown	0	1 (1)	1 (1)	
Nodal stage				*P* = .69
N0	97 (67)	88 (69)	104 (70)	
N1	38 (27)	29 (23)	27 (18)	
N2-N3	9 (6)	9 (7)	8 (5)	
Unknown	0 (0)	1 (1)	11 (7)	
Treatment type				p = 0.96
Surgery	109 (76)	98 (77)	114 (76)	
NACT^§^	35 (24)	29 (23)	36 (24)	
Time to treatment (median in days)	44	37	43	

^*^Numbers in brackets are percentages.

^§^NACT: neo-adjuvant chemotherapy.

^†^POC: point of contact.

The most common POC was a surgeon, regardless of the year: 61% in 2019, 72% in 2020, and 58% in 2021. For the same years, a radiologist served as POC for 35%, 23%, and 37% of the patients. Although these differences were not statistically significant, the absolute number of patients first seen by a surgeon was similar in 2019 and 2021, around 90, while patients referred to a radiologist in 2020 (29) were around half of those in 2019 or 2021 (51 and 55, respectively) (Table 1).

**Figure 2.**
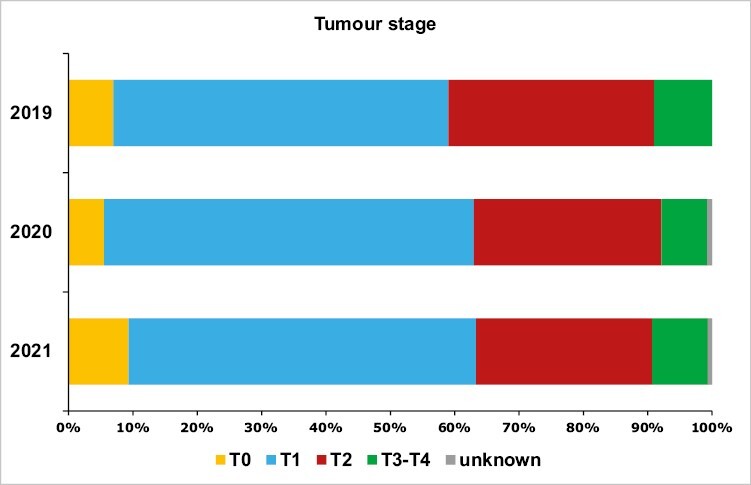
Distribution by tumor stage.

The distribution of patients by tumor stage was substantially unchanged over time. The proportion of patients with a tumor of 2 cm or less was in the range 52%-57%, while the proportion of those presenting with a tumor from 2.1 up to 5 cm was in the range 27%-32%. Patients affected by in situ tumors, or with tumors larger than 5 cm or locally advanced, represented less than 10% of the patient population. Of note, in situ tumors were numerically slightly fewer in 2020 (7), compared to 2019 or 2021 (10 and 14, respectively; Table 1; Fig. 2).

**Figure 3.**
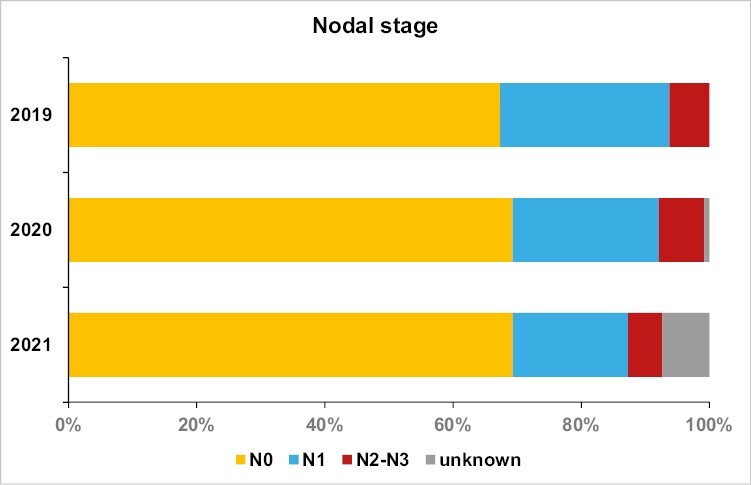
Distribution by nodal stage.

Similarly, nodal stage distribution was similar across the whole study period. Patients were mostly node-negative, with proportions varying between 67% and 70%. The proportion of patients with one to 3 lymph nodes was in the range 18%-27%. Patients with 4 or more lymph nodes accounted for less than 10% of the population (Table 1; Fig. 3).

The distribution by type of first curative treatment was broadly similar in 2019, 2020, and 2021. The proportion of patients receiving upfront surgery was 77% in 2020, compared to 76% in 2019 and 2021.

The odds ratio for treatment delay (45 days or more) was 0.87 for 2020 versus 2019 (95% CI, 0.5-1.53) and 0.9 for 2021 versus 2019 (95% CI, 0.52-1.55), after adjusting for type of POC, presentation with symptoms, treatment type, tumor stage, nodal stage, and EBC subtype (ie, luminal, HER2-positive, and triple-negative).

## Discussion

We conducted a cross-sectional study to assess the impact of the COVID-19 pandemic on diagnostic and care pathways in a large tertiary-level cancer center in Italy, using granular tumor, patient, and treatment-related data. To our knowledge, this is the largest study of this kind for Italy, which was hardly hit by the pandemic as soon as in March 2020.

In our study, there was no evidence of major changes in the management of EBC patients during 2019-2021 and no treatment delays were observed.

However, we found a slight decrease in the absolute number of patients being treated in 2020, offset by an increase in 2021 to levels comparable to 2019. This finding is in line with results from other reports, which may reflect patients’ decision to avoid seeking care due to the perceived risk of infection or the restrictions in mobility during lockdowns.^12,13^

Our study suggests that disruption of breast cancer screening programs may have impacted on the characteristics of the patient population, with a larger proportion of women presenting with palpable nodules. Our data are in line with the findings from a nationwide English study, suggesting that around 2500-4100 cancers would shift from screen-detected to symptomatic cancers.^14^ Findings from our study also mirror those from Hawrot et al, who conducted a hospital-based, retrospective study including patients diagnosed with EBC between January and May 2020 in Philadelphia. They found that a higher proportion of breast cancer was detected by self-palpation in 2020 compared with historical cohorts.^15^

The implementation of screening programs for early detection of breast cancer have resulted in a remarkable reduction in breast cancer mortality.^16-20^ The suspension of breast cancer screening during the COVID-19 pandemic raised concerns that the delay in diagnosis and treatment would lead to an excess of breast cancer deaths in the coming years. Modelling studies predicted 10 000 excess deaths from breast or colorectal cancer in the next decade because of cancer screening halt.^21^ According to a cancer microsimulation modelling tool created by The Canadian Partnership Against Cancer, a 3-month pause in cancer screening and diagnosis could potentially lead to an excess of 310 patients being diagnosed at advanced stage and an excess of 110 cancer-related deaths between 2020 and 2029.^22^ Sud et al predicted that a 3-month delay in diagnosis would potentially result in an up to 7.7% reduction in 10-year survival.^23^ It will take time to assess the true impact of screening activity suspension on survival from breast cancer.

Here there was no evidence of major changes in the distribution of patients by tumor or nodal stage during 2019-2021. Our results are not consistent with those from other studies, which found that more women were diagnosed with breast cancer at more advanced stages during the COVID-19 pandemic, compared topre-pandemic figures.^22,24-28^ Conversely, results from Tonneson et al are in line with our findings. The study compared the stage distribution of new breast cancer diagnoses at the Mayo Clinic (Rochester, Minnesota) from March 2020 to August 2020 with those from March 2019 to August 2019. There was no evidence for major changes in disease parameters.^29^

The main limitation of our study is the cross-sectional, mainly descriptive, study design. Moreover, this study was conducted in a single cancer center, with dedicated cancer services. Even if all patients presenting with newly diagnosed breast cancer in the breast unit catchment area were fully ascertained, we cannot rule out that our findings may not be representative of the population at large.

Validation on a larger, population-based cohort of patients is warranted to robustly assess the impact of the COVID-19 pandemic on treatment practices and outcomes for patients with EBC.

## Data Availability

The data underlying this article will be shared on reasonable request to the corresponding author.
